# ERK3 is transcriptionally upregulated by ∆Np63α and mediates the role of ∆Np63α in suppressing cell migration in non-melanoma skin cancers

**DOI:** 10.1186/s12885-021-07866-w

**Published:** 2021-02-12

**Authors:** Eid S. Alshammari, Amjad A. Aljagthmi, Andrew J. Stacy, Mike Bottomley, H. Nicholas Shamma, Madhavi P. Kadakia, Weiwen Long

**Affiliations:** 1 Department of Biochemistry and Molecular Biology, Boonshoft School of Medicine, Wright State University, 112 Diggs Laboratory, 3640 Colonel Glenn Highway, Dayton, OH 45435 USA; 2 Department of Clinical Laboratory Sciences, College of Applied Medical Sciences, Jouf University, Sakakah, 72388 Saudi Arabia; 3 Department of Math and Microbiology, College of Science and Mathematics, Wright State University, Dayton, OH 45435 USA; 4 Department of Dermatology, Boonshoft School of Medicine, Wright State University, 3640 Colonel Glenn Highway, Dayton, OH 45435 USA

**Keywords:** ΔNp63α, ERK3, IHC, SCC, NMSC, Migration

## Abstract

**Background:**

p63, a member of the p53 gene family, is an important regulator for epithelial tissue growth and development. ∆Np63α is the main isoform of p63 and highly expressed in Non-melanoma skin cancer (NMSC). Extracellular signal-regulated kinase 3 (ERK3) is an atypical mitogen-activated protein kinase (MAPK) whose biochemical features and cellular regulation are distinct from those of conventional MAPKs such as ERK1/2. While ERK3 has been shown to be upregulated in lung cancers and head and neck cancers, in which it promotes cancer cell migration and invasion, little is known about the implication of ERK3 in NMSCs.

**Methods:**

Fluorescent immunohistochemistry was performed to evaluate the expression levels of ΔNp63α and ERK3 in normal and NMSC specimens. Dunnett’s test was performed to compare mean fluorescence intensity (MFI, indicator of expression levels) of p63 or ERK3 between normal cutaneous samples and NMSC samples. A mixed effects (ANOVA) test was used to determine the correlation between ΔNp63α and ERK3 expression levels (MFI). The regulation of ERK3 by ΔNp63α was studied by qRT-PCR, Western blot and luciferase assay. The effect of ERK3 regulation by ΔNp63α on cell migration was measured by performing trans-well migration assay.

**Results:**

The expression level of ∆Np63α is upregulated in NMSCs compared to normal tissue. ERK3 level is significantly upregulated in AK and SCC in comparison to normal tissue and there is a strong positive correlation between ∆Np63α and ERK3 expression in normal skin and skin specimens of patients with AK, SCC or BCC. Further, we found that ∆Np63α positively regulates ERK3 transcript and protein levels in A431 and HaCaT skin cells, underlying the upregulation of ERK3 expression and its positive correlation with ∆Np63α in NMSCs. Moreover, similar to the effect of ∆Np63α depletion, silencing ERK3 greatly enhanced A431 cell migration. Restoration of ERK3 expression under the condition of silencing ∆Np63α counteracted the increase in cell migration induced by the depletion of ∆Np63α. Mechanistically, ERK3 inhibits the phosphorylation of Rac1 G-protein and the formation of filopodia of A431 skin SCC cells.

**Conclusions:**

ERK3 is positively regulated by ∆Np63α and mediates the role of ∆Np63α in suppressing cell migration in NMSC.

**Supplementary Information:**

The online version contains supplementary material available at 10.1186/s12885-021-07866-w.

## Background

ΔNp63α is the predominant and physiologically significant isoform of p63 in epithelial tissues [1–3]. ΔNp63α is highly expressed in the basal layer of the epidermal keratinocytes [1, 3, 4]. As ΔNp63α is a critical regulator in epithelial development, alterations in its expression and functions are implicated in tumor development. While its protein expression varies in a different type of tumors, it is highly expressed in squamous cell carcinoma (SCC) and basal cell carcinoma (BCC) of diverse tissue origins, particularly in the skin and lung [5, 6].

ΔNp63α exhibits various functions in tumorigenesis of different types of cancers [7]. ΔNp63α is upregulated and shown to be a biomarker for non-invasive epithelial cancers, whereas it is undetectable in the invasive adenocarcinomas of the prostate, breast, and colon [8, 9]. ΔNp63α promotes tumor initiation by activating signaling pathways involved in cell survival. For example, ΔNp63α promotes the activation of AKT pathway, which in turn enhances cell proliferation of pancreatic cancer [10, 11] and squamous cell carcinoma of the lung [12]. Premalignant lesions and epidermal cysts were observed in the basal layer of the epidermis upon ΔNp63α induction, indicating an oncogenic role for ΔNp63α in the initiation of SCC tumorigenesis [13]. Nevertheless, an inhibitory effect of ΔNp63α on cancer cell invasion has been reported in breast, bladder, and prostate cancers [14–16]. These findings indicate the complexity of ΔNp63α’s involvement in different types of cancers, and therefore further studies need to be done to elucidate how ΔNp63α plays differential roles in different tumors.

Extracellular signal-regulated protein kinase 3 (ERK3), also known as MAPK6, is atypical MAPK [17]. In contrast with the well-studied classical MAPKs ERK1/2, much less is known about the regulation of ERK3 signaling. Several studies have shown that ERK3 is upregulated in multiple cancers [18–20]. Our previous study demonstrated that ERK3 mRNA levels are seven-fold higher in squamous cell lung carcinoma when compared to healthy lung tissue [18]. In addition, the protein level of ERK3 is upregulated in 65% of non-small cell lung carcinomas, with higher expression in squamous cell lung carcinoma than lung adenocarcinoma as determined by tumor tissue microarray [18]. In oral squamous cell carcinoma, ERK3 mRNA level was five-fold higher than the normal mouth tissue [20]. Another study showed a four-fold increase in ERK3 protein level in gastric cancer tissue [19]. In line with its upregulation in cancers described above, ERK3 promotes migration and invasion of lung cancer cells [18], breast cancer cells [21] and head and neck cancer cells [22]. On the other hand, ERK3 also has been shown to play inhibitory roles in growth and migration of some cancer cells. ERK3 inhibits melanoma cell proliferation and migration/invasion [23, 24] and suppresses intrahepatic cholangiocarcinoma cell growth both in vitro and in vivo in xenograft mice [25].

Unlike ΔNp63α whose regulation and role in NMSC have been investigated, the involvement of ERK3 signaling in NMSC has not been reported. In this study, we showed that both ΔNp63α and ERK3 levels are upregulated and highly positively correlated in NMSC. We also showed that ΔNp63α directly upregulates ERK3 gene transcription and expression levels in NMSC and that ERK3 mediates the role of ΔNp63α in regulating cancer cell migration.

## Methods

### Cell culture and reagents

The squamous cell carcinoma cell line A431 was purchased from American Type Culture Collection (Manassas, Virginia, USA) while the non-tumorigenic immortalized human keratinocyte HaCaT cell was obtained from Dr. Nancy Bigley (Wright State University). A431 and HaCaT cell lines were cultured in Dulbecco’s Modified Eagle’s medium (DMEM) supplemented with 10% fetal bovine serum (FBS) and 1% Penicillin-Streptomycin.

### siRNA transfection, transient transfection and lentiviral transduction

siRNA transfection was conducted using Dharma-FECT transfection reagent (Dharmacon, Lafayette, CO, USA) or Lipofectamine RNAi-Max (ThermoFisher Scientific, Carlsbad, CA, USA), following the manufacturer’s instructions. ERK3 siRNA was purchased from Ambion/ThermoFisher Scientific (siERK3, cat # 4390824, assay ID s11148) and ΔNp63α siRNA or non-silencing control (NSC) were purchased from Qiagen (Valencia, CA, USA) as previously described [26].

For transient transfection, A431 cells were transfected with empty vector control (EV) or ERK3 plasmids using Lipofectamine 3000 reagent (Thermo Fisher Scientific, Carlsbad, CA, USA), and 48 h after transfection cells were harvested and tested for protein expression. A431 cells were transduced with lentiviruses expressing an empty vector CDH or CDH-ERK3 as previously described [27].

### Immunoblot analysis

Cells were lysed in the lysis buffer containing 50 mM Tris (pH 7.5), 150 mM NaCl, 0.5% NP-40, 1 mM complete protease inhibitors and 1 mM phosphatase inhibitor cocktail III (Sigma-Aldrich). Immunoblot analysis was carried as previously described [27]. The following primary antibodies were used in immunoblotting: Mouse monoclonal anti-pan-p63 (4A4), rabbit monoclonal anti-ERK3 (ab53277, 1:1000, Abcam), rabbit polyclonal anti-pRac1 S71 (sc-12924, 1:500, Santa Cruz), mouse monoclonal anti-Rac1(ab33186, 1:5000, Abcam) and mouse monoclonal anti-β-actin (A5316, 1:20,000, Sigma-Aldrich) antibodies were used to detect ΔNp63α, ERK3, pRac1, Rac1 and β-actin, respectively. β-actin was used as a loading control. Appropriate secondary antibodies (HRP-conjugated goat anti- mouse [170-6516, Biorad] or anti-rabbit [170-6515, Biorad]) were used for visualization by chemiluminescence (Thermo Scientific).

### RNA extraction and RT-qPCR

Total RNA was extracted from cells 24-h post-transfection for gene expression analysis using Trizol reagent (Ambion). SuperScript VILO Master Mix (Invitrogen) was used for reverse transcription to generate cDNA as per the manufacturer’s instructions. Quantitative Polymerase chain reaction (qPCR) was performed using TaqMan® Universal Master Mix (Applied Biosystems), designed Roche Universal primers and Universal Probe (Roche Diagnostics) on the 7900HT Fast Real-Time PCR Systems (Applied Biosystems) using the following primers: GAPDH forward primer [AGCCACATCGCTCAGACAC], GAPDH reverse primer [GCCCAATACGACCAAATCC], ERK3 forward primer [TTTGCTGAAATGCTGACTGG], and ERK3 reverse primer [CCAGTCAGCATTTCAGCAAA]. GAPDH was used as internal control, and the relative gene expression level was calculated by the ΔΔCT method [28].

### Trans-well migration assay

Cell migration was analyzed using a modified two chamber trans-well system (8.0 μm pore, BD Biosciences Falcon) according to the manufacturer’s protocol. At 24 h post-transfection, cells were detached by trypsin/EDTA, washed once with serum-free medium, and then re-suspended in serum-free medium. 0.6 mL of complete medium with 10% FBS was added to the bottom of each well. A total of 1.5 × 10^6^ cells per well was added in trans-well inserts and cells allowed to migrate for 18–20 h in a 37 °C incubator. Using cotton swabs, cells which failed to migrate in the upper surface of the trans-well were removed. The migrated cells attached on the undersurface of inserts were fixed with 10% formalin, stained with crystal violet solution (0.5% in water) and followed by quick washes with distilled water or PBS. Migrated cells were then photographed under a microscope at 10 X magnification, and five images per condition were taken to count the migrated cells by ImageJ 1.52 software.

### Cloning of the ERK3 reporter and luciferase reporter assay

The fragments of ERK3 gene enhancer region containing p63 binding sites was amplified by PCR using the following primers: BS1 forward primer [GCGCGGTACCGTTCTTCTTTGTTTCCTCAG], BS1 reverse primer [GCCACTCGAGCACGTTCAAACCATAGCAAC], BS2 forward primer [GCGGTACCAGGTCTTAGTGCTGTTGTAG] and BS2 reverse primer [GCGTCTCGAGCCTAAACACTATGCAATGCTG] and cloned into the PGL3-promoter luciferase vector via KPN1 and XHO1. H1299 cells were plated on 24-well plates and co-transfected with p63-BS1 or –BS2 luciferase reporters and Renilla luciferase constructs along with empty vector control or 0.1 or 0.3 μg of ΔNp63α. The luciferase assay was performed as previously described [29].

### Tissue immunofluorescence staining

Formalin-fixed, paraffin-embedded human skin tissue microarrays described previously [6] were used for co-immunostaining studies. Human tissue samples consisted of normal skin (*N* = 53), actinic keratosis (AK) (*N* = 66), cutaneous squamous cell carcinoma (SCC) (*N* = 59), and basal cell carcinoma of the skin (BCC) (*N* = 57). Skin tumor and non- tumor skin tissue slides were co-stained for p63 and ERK3 as previously described with some modifications [6, 10]. Briefly, the staining of both p63 and ERK3 were performed using heat-based antigen retrieval processes with a citrate buffer (10 mM sodium citrate, 0.05% Tween-20, pH 6.0). Tissues were incubated with anti-p63 (4A4, 1:800) and anti-ERK3 (ab53277, 1:50, Abcam) primary antibodies at 4 C. Tissue sections were visualized and imaged using a Leica CTR 6000 Microscope (Leica Microsystems, Wetzlar, Germany) and ImagePro 6.2 software (Media Cybernetics, Bethesda, MD). Three representative pictures were taken under a microscope at 20 X magnification for each tissue sample to measure the mean fluorescence intensity (MFI). Background intensity first was subtracted, and then nine measurements of fluorescence intensity at 9 different areas with the same size were taken for each tissue sample. Average mean fluorescence intensity was calculated using ImageJ 1.52 software.

### Cell immunofluorescence

A431 cells transduced with CDH or CDH-HA-ERK3 lentiviral vectors were grown on sterile glass coverslips, and at 24-h post plating, cells were washed with 1xPBS prior fixation with 4% paraformaldehyde for 15 min. After three washes with 1xPBS for 5 min, cells were permeabilized with 0.2% triton- X-100 for 5 min followed by three washes with 1XPBS for 5 min. Cells were blocked with 5% normal goat serum in 1XPBS for 1 h at room temperature and then incubated with mouse monoclonal anti-HA antibody (Sigma-aldrich, MO, USA) for overnight at 4 C. After three washes with 1XPBS for 5 min, cells were incubated with Alexa Fluor 555 Phalloidin (1:40, Invitrogen, Carlsbad, CA, USA) and Alexa Fluor goat anti-mouse 488 (1:500, Invitrogen, Carlsbad, CA, USA) for 90 min at room temperature. Cells were washed with 1XPBS for four times 5 min each, and mounted with Vecta-Shield plus DAPI Mounting media (Vector Laboratories, Burlingame, CA, USA). Cells were visualized and imaged at 63 X magnification using a Leica CTR 6000 Microscope (Leica Microsystems, Wetzlar, Germany) and ImagePro 6.2 software (Media Cybernetics, Bethesda, MD).

### Generation of stable cell lines expressing ERK3 shRNA by lentiviral transduction

A431 and HaCaT cell lines with stable knockdown of endogenous ERK3 were generated by lentiviral expression of a short hairpin RNA (shRNA) specifically targeting ERK3 mRNA (shERK3) in the presence of 5 μg/ml polybrene. As a control, cells with stable expression of the non-targeting shRNA (shGIPZ) were used. Cells were split and selected 2 days post-transduction by puromycin (0.8 μg /mL for A431, 1 μg /mL for HaCaT) for 14 days. The knockdown was confirmed by Western blotting analysis and RT-qPCR.

### Cell proliferation assay

Cell proliferation was determined using the CellTiter 96® AQueous One Solution Cell Proliferation Assay Kit (Promega, Madison, WI), following the manufacturer’s instructions. Stable A431 or HaCaT cells were plated in five 96-well plates and allowed to grow at 37 °C in the incubator. At specific time points, MTS-containing reagent was added to the cells. After 2 h incubation, the absorbance was measured by Synergy H1 microplate reader (BioTek, Winooski, VT) at 490 nm.

### Statistical analysis for stained tissue

Adjusted mean fluorescence intensity and standard error of mean (SEM) levels of p63 and ERK3 from all type of skin tissue (normal skin samples, basal cell carcinoma samples, squamous cell carcinoma samples and actinic keratosis samples) were plotted. A mixed effects (ANOVA) tests were used to determine the correlation between the two response variables (MFI of p63 and ERK3) in nine measurements per sample. Post-hoc multiple comparison methods using Dunnett’s test were performed to compare between MFI values of p63 or ERK3 between control samples (i.e., normal skin samples) and all other samples (i.e., AK, SCC, BCC) (Dunnett, 1955, 1980). PROC MIXED procedure (SAS/STAT®, Ver 9.4, SAS Institute Inc., Cary, NC) was used for analyses [30, 31]. A *P-*value of less than 0.05 was considered statistically significant.

## Results

### ERK3 expression is upregulated in NMSC and positively correlated with ΔNp63α expression in normal skin and NMSC

**Fig. 1:**
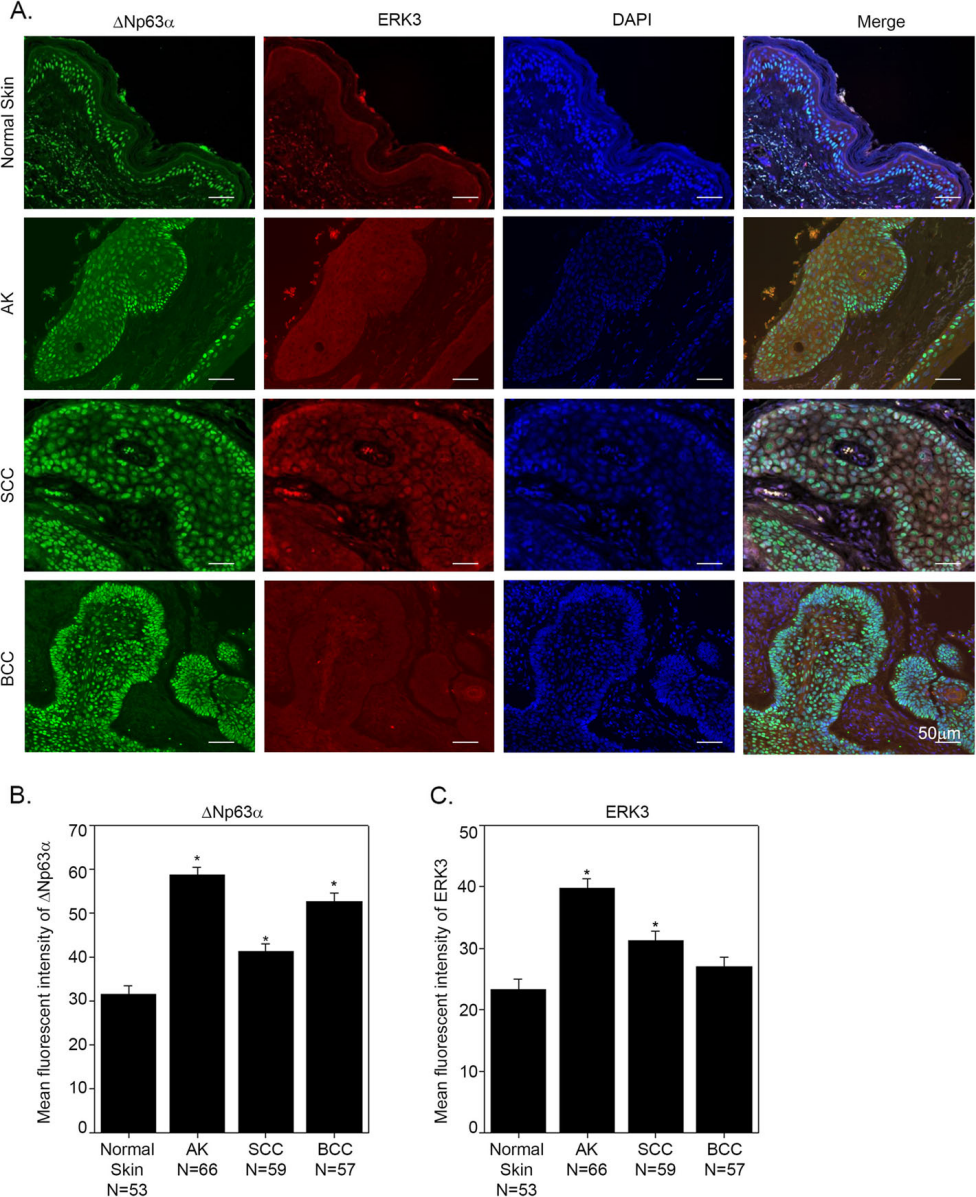
ΔNp63α and ERK3 are highly expressed in NMSC. **a** Representative images of FFPE human skin tissue microarrays were co-immunostained for ΔNp63α and ERK3 in normal skin, AK, SCC and BCC (scale bar = 50 μm). Quantitation of ΔNp63α and ERK3 levels from 53 normal skin samples, 66 AK, 59 SCC and 57 BCC samples are plotted. Y-axis represents the mean fluorescent intensity. Bar plots showing least squares means + SE for ΔNp63α (**b**) and ERK3 (**c**). Significance differences indicated at *P-*value < 0.05 (*) and *P-*value < 0.01(**) in comparison with normal skin by Student’s t-test

**Fig. 2:**
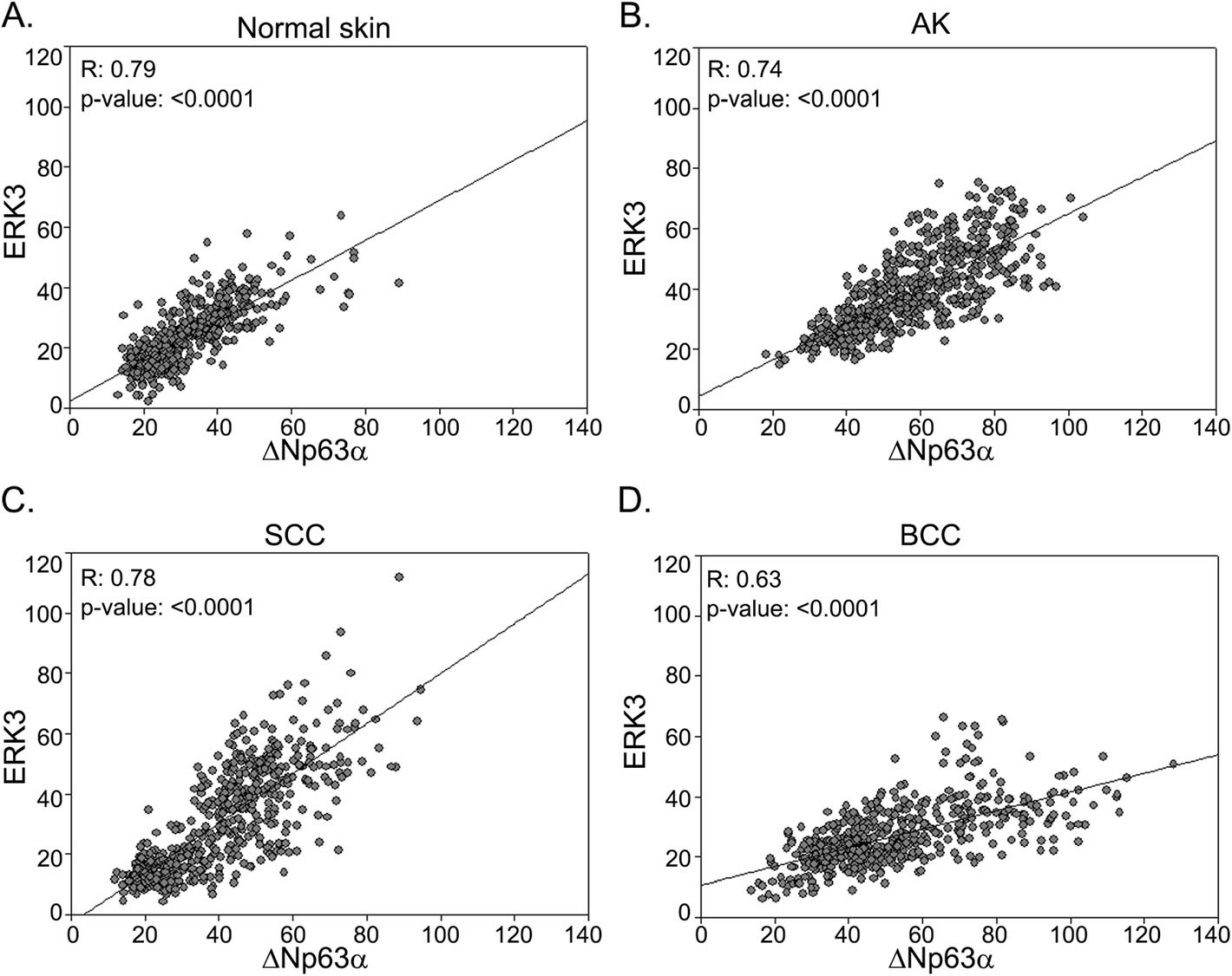
ΔNp63α and ERK3 levels positively correlate in normal skin and NMSC. The mean fluorescence intensity of ΔNp63α protein and ERK3 protein were plotted for each skin tissue type. X and Y-axes represents the mean fluorescence intensity for ΔNp63α and ERK3, respectively, for **a** normal, **b** AK, **c** SCC and **d** BCC specimens. *P-*value was calculated using Student’s t-test

ΔNp63α has been previously shown to be upregulated in NMSC. While ERK3 is shown to be upregulated in multiple cancers including squamous cell lung carcinomas [18] and head and neck cancers [22], little is known about its involvement in NMSC. We wanted to investigate ERK3 expression levels and its association with ΔNp63α expression at specific stages in the progression of normal skin to SCC and BCC. We examined the expression levels of ΔNp63α and ERK3 proteins in formalin-fixed, paraffin-embedded (FFPE) human skin tissue microarray sections. Representative images of the histology of the normal skin, AK, SCC and BCC are shown in Additional file 1: Figure S1. Skin tissue microarrays used in this study included normal skin (*N* = 53), a precursor to squamous cell carcinoma/actinic keratosis (AK) (*N* = 66), cutaneous squamous cell carcinoma (SCC) (*N* = 59) and cutaneous basal cell carcinoma (BCC) (*N* = 57) sections (Additional file 7: Table S1). Consistent with a previous report from our lab [6], ΔNp63α is significantly upregulated in AK, SCC and BCC skin tissues compared with normal skin (Fig. 1a and b and Additional file 8: Table S2). ERK3 protein was also shown to be expressed in all four types of skin sections and mainly localized to the cytoplasm of the cells (Fig. 1a and c and Additional file 8: Table S2). Similar to the expression status of ΔNp63α, ERK3 is significantly upregulated in AK and SCC cutaneous tissues as compared to normal skin tissues (Fig. 1b and c). However, there is no statistically significant difference in ERK3 expression between BCC and normal skin (Fig. 1c). We next examined whether there is a correlation between the expression of ΔNp63α and ERK3 in AK and SCC specimens when compared to normal skin tissues (Fig. 1). We found a significant positive correlation between ΔNp63α and ERK3 protein levels in all four tissue types of skin (*P*-values are < 0.0001) (Fig. 2a-d and Additional file 9: Table S3). This indicates that ΔNp63α might positively regulate ERK3 in normal skin and NMSC.

### ΔNp63α positively regulates ERK3 expression

**Fig. 3:**
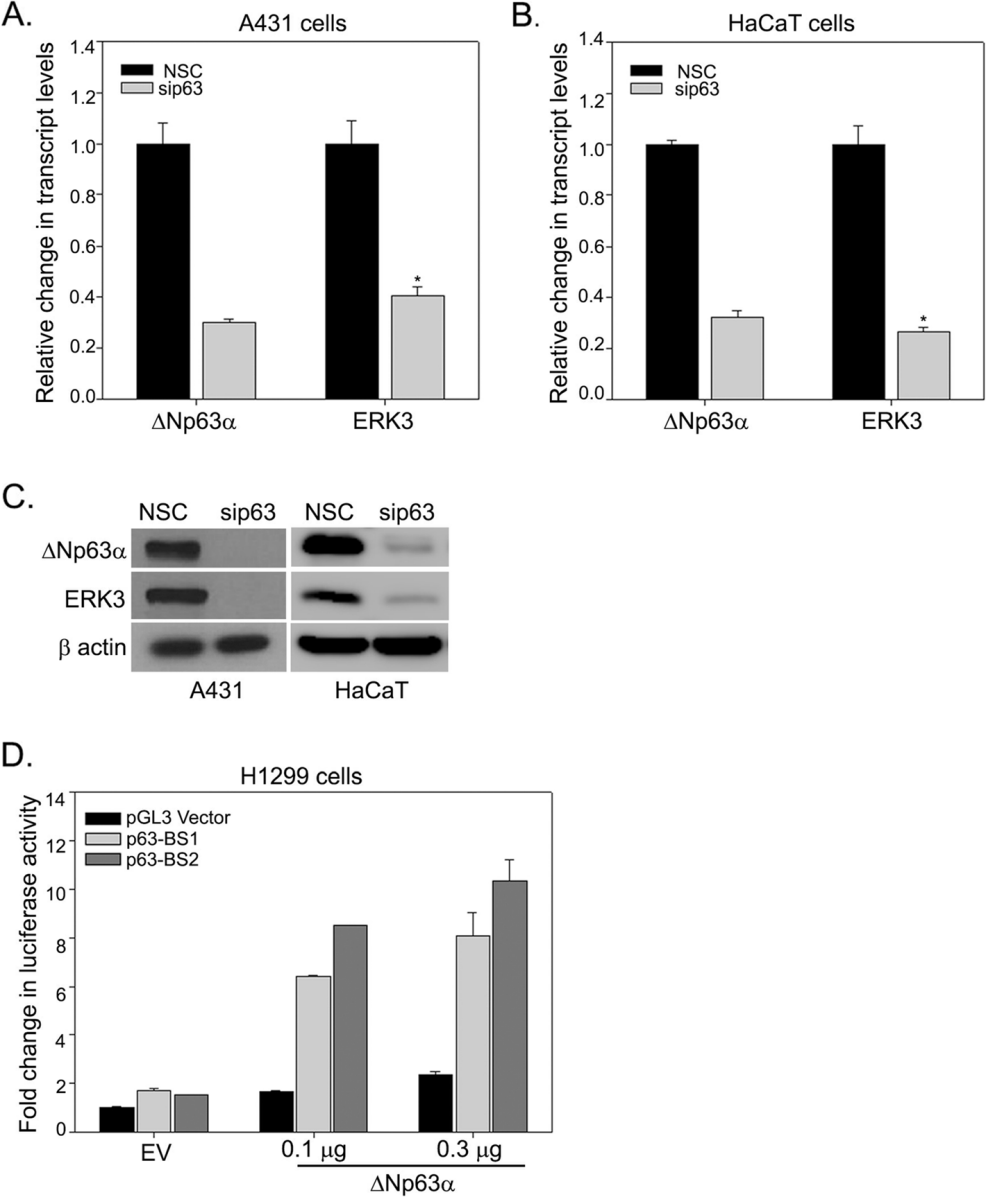
ΔNp63α positively regulates ERK3 gene expression. **a** A431 and **b** HaCaT cells were transiently transfected with non-silencing control siRNA (NSC) or siRNA specific to p63 (sip63). Total RNA was extracted and ΔNp63α and ERK3 transcript levels were measured by qRT-PCR. Values represent mean ± S.D. of three experiments. An asterisk indicates a significant difference with *P* < 0.05 by Student’s t-test. **c** Immunoblots of ΔNp63α and ERK3 in A431 and HaCaT cells transfected with NSC or sip63. Immunoblot with β-actin was performed to confirm equivalent protein loading. Representative western blots are shown as cropped gel images. Full length blots are presented in Additional File 2 (Figure S2). **d** H1299 cells were co-transfected with pGL3 empty vector, p63-BS1- or p63-BS2-Luc reporter constructs along with the empty vector (EV) or 0.1 μg or 0.3 μg of ΔNp63α expressing plasmid. Cells were subjected to dual luciferase assay at 24 h post-transfection. The Y-axis represents the fold change in the luciferase activity compared to cells co-transfected with pGL3 empty vector and empty vector control (EV)

Since both ΔNp63α and ERK3 are upregulated and positively correlated in NMSC tissues, we examined whether ΔNp63α upregulates the expression levels of ERK3. We silenced ΔNp63α in A431 squamous cell carcinoma cells and HaCaT non-tumorigenic keratinocyte cells, both of which primarily express the ΔNp63α isoform of p63 [26]. We observed that silencing ΔNp63α resulted in a significant reduction of ΔNp63α at both transcript and protein levels in both cell lines as expected (Fig. 3a-c). Importantly, ERK3 transcript level was significantly reduced upon silencing of ΔNp63α in A431 and HaCaT cell lines (Fig. 3a and b). Consistent with the change in mRNA transcript level, immunoblot analysis shows that ERK3 protein expression was also substantially decreased upon ΔNp63α silencing (Fig. 3c). These findings suggest that ΔNp63α positively regulates ERK3 levels in normal skin and SCC cells.

To investigate how ΔNp63α regulates the expression of ERK3, we looked into a previous p63 ChIP-seq study (GSE59827), in which we found two putative p63 binding sites within the enhancer region of ERK3 [32, 33]. The first binding site (BS1) is 15 kb (chr15:52004994-52005013) upstream and the second site (BS2) is 3.4 kb (chr15:52069703-52069722) downstream the transcription start site (TSS) of the ERK3 gene. To confirm that ΔNp63α directly targets ERK3, we cloned these two regions containing p63 binding sites into the pGL3-promoter luciferase vector. We next co-transfected H1299 cells with pGL3 empty vector, p63-BS1- or p63-BS2-luc reporters along with empty vector control or increasing amount of ΔNp63α expression plasmid. ΔNp63α expression induced a dose-dependent increase in p63-BS1 and p63-BS2 luciferase activity (Fig. 3d). Taken together, these results suggest that ERK3 is a direct transcriptional target of ΔNp63α.

### ERK3 silencing enhances cell migration

**Fig. 4:**
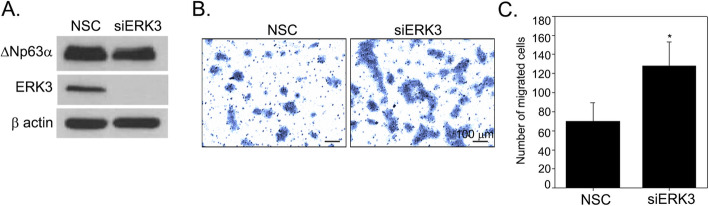
Silencing ERK3 enhances cell migration of A431 cells. **a** A431 cells were transiently transfected with either non-silencing control siRNA (NSC) or ERK3 siRNA (siERK3) as indicated. Two days post-transfection, one set of cells were harvested, and the change in proteins level was analyzed by immunoblot analysis. Representative western blots are shown as cropped gel images. Full length blots are presented in Additional File 3 (Figure S3). The other set of cells were used for the transwell cell migration assay, and the number of migrated cells was quantitated after 18 h. Representative images of migrated cells with crystal violet staining were shown in (**b**). The average number of migrated cells per well is presented in the y-axis of the bar graph in (**c**). Values represent mean ± S.D. An asterisk indicates a significant difference with *P-*value < 0.0001 by Student’s t-test

ΔNp63α has an inhibitory role on cell migration and invasion as it downregulates several genes that promote epithelial-to-mesenchymal transition and cancer cell invasion and metastasis [11, 26, 29, 34, 35]. In addition, the inhibitory effect of ERK3 on cell migration in tongue squamous cell carcinoma cell line by downregulating Rac1 level was also reported [36]. To determine the role of ERK3 in skin cancer cell migration, A431 cells were transfected with siRNA targeting ERK3 or non-silencing control. Western blotting analysis showed a significant reduction in ERK3 protein levels confirming ERK3 silencing (Fig. 4a). The depletion of ERK3 led to a significant increase in the migration of A431 cells when measured by trans-well migration assay (Fig. 4b and c). These results suggest that similar to ΔNp63α, ERK3 suppresses cancer cell migration of cutaneous SCC cells.

### ERK3 mediates the inhibitory role of ΔNp63α in suppressing cancer cell migration

**Fig. 5:**
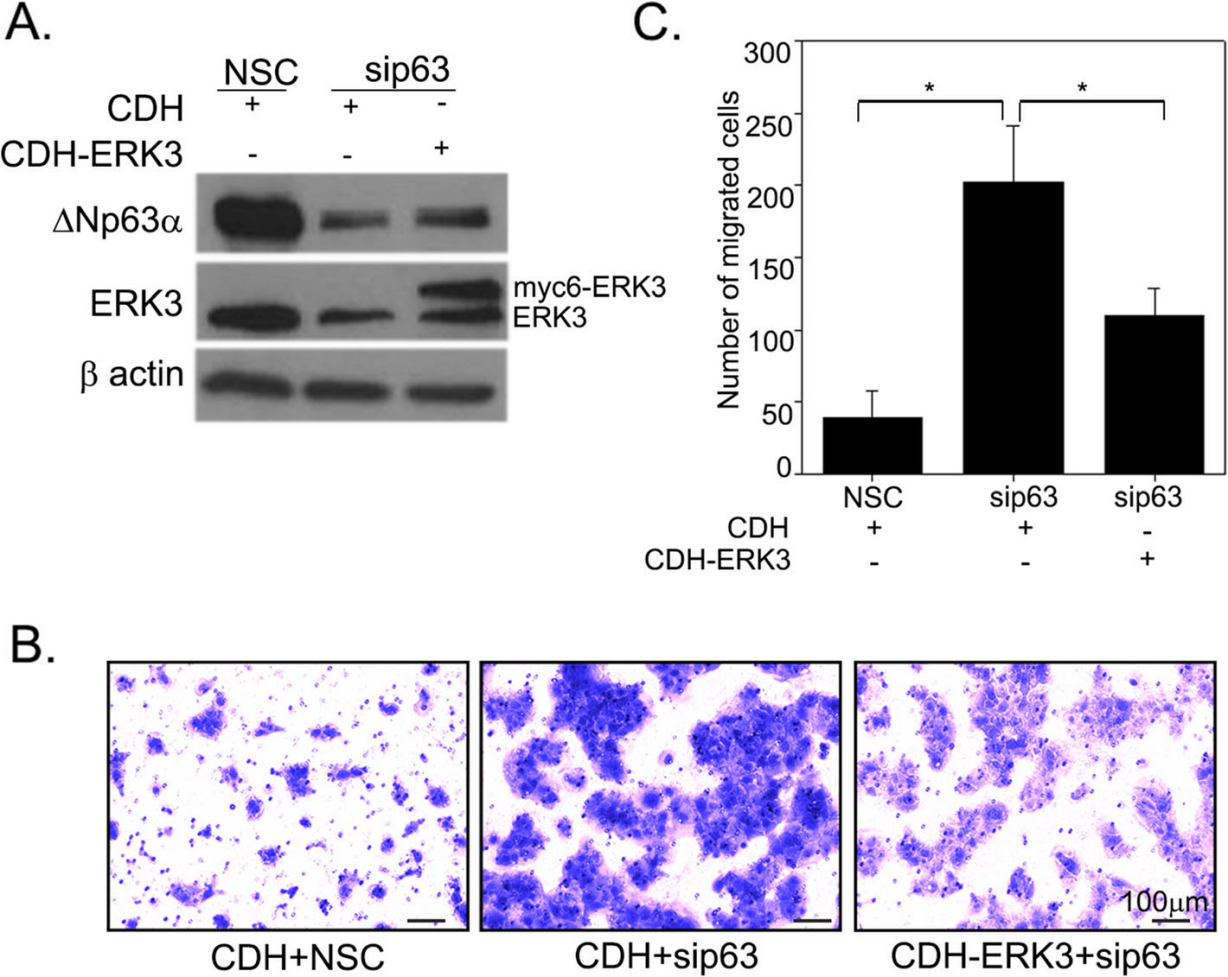
ERK3 restoration counteracts the increase in cell migration induced by ΔNp63α silencing. **a** A431 cells were transiently transfected for two rounds with non-silencing control siRNA (NSC) or ΔNp63α siRNA (sip63) as indicated. Along with the second sip63 transfection, cells were also transduced with lentiviral empty vector pCDH CMV-MCS-EF1-Puro (CDH) or pCDH-Myc6-ERK3 (CDH-ERK3) as indicated. Twenty-four hours after lentivirus transduction, one set of cells were harvested, and ERK3 and ΔNp63α proteins level were analyzed by immunoblot analysis. Exogenously expressed ERK3 protein with 6 myc-tags (Myc6-ERK3, the upper bands) and endogenous ERK3 protein (lower band) were indicated by arrows. Representative western blots are shown as cropped gel images. Full length blots are presented in Additional File 4 (Figure S4). The other set of cells were subjected to trans-well cell migration assay and the number of migrated cells was quantitated after 18 h. Representative images of migrated A431 cells with crystal violet staining under each condition were shown in (**b**). The average number of migrated cells per well is presented in the y-axis of the bar graph in (**c**). Values represent mean ± S.D. An asterisk indicates a statistically significant difference with *P* < 0.0001 by Student’s t-test

As we have found that ΔNp63α upregulates ERK3 expression and that both ΔNp63α and ERK3 suppress cell migration of A431 cells, we examined whether ERK3, as a downstream target of ΔNp63α, mediates the latter’s role in cell migration. To test this, we silenced ΔNp63α in A431 cells with siRNA against ΔNp63α concomitant with ERK3 expression rescue by lentiviral expression of ERK3 cDNA (CDH-ERK3). As expected, ΔNp63α silencing greatly decreased the protein level of ERK3 (Fig. 5a) and led to a significant increase in the number of migrated cells (Fig. 5b and c). Importantly, restoration of ERK3 protein level by lentiviral expression of exogenous myc-tagged ERK3 (Fig. 5a) significantly decreased cell migration induced by ΔNp63α silencing (compare “CDH + sip63” with “CDH-ERK3 + sip63” in Fig. 5b and c). These findings suggest that ERK3 is an important downstream mediator of ΔNp63α in suppressing A431cell migration.

### The depletion of ERK3 does not affect the cell proliferation of HaCaT or A431 cells

**Fig. 6:**
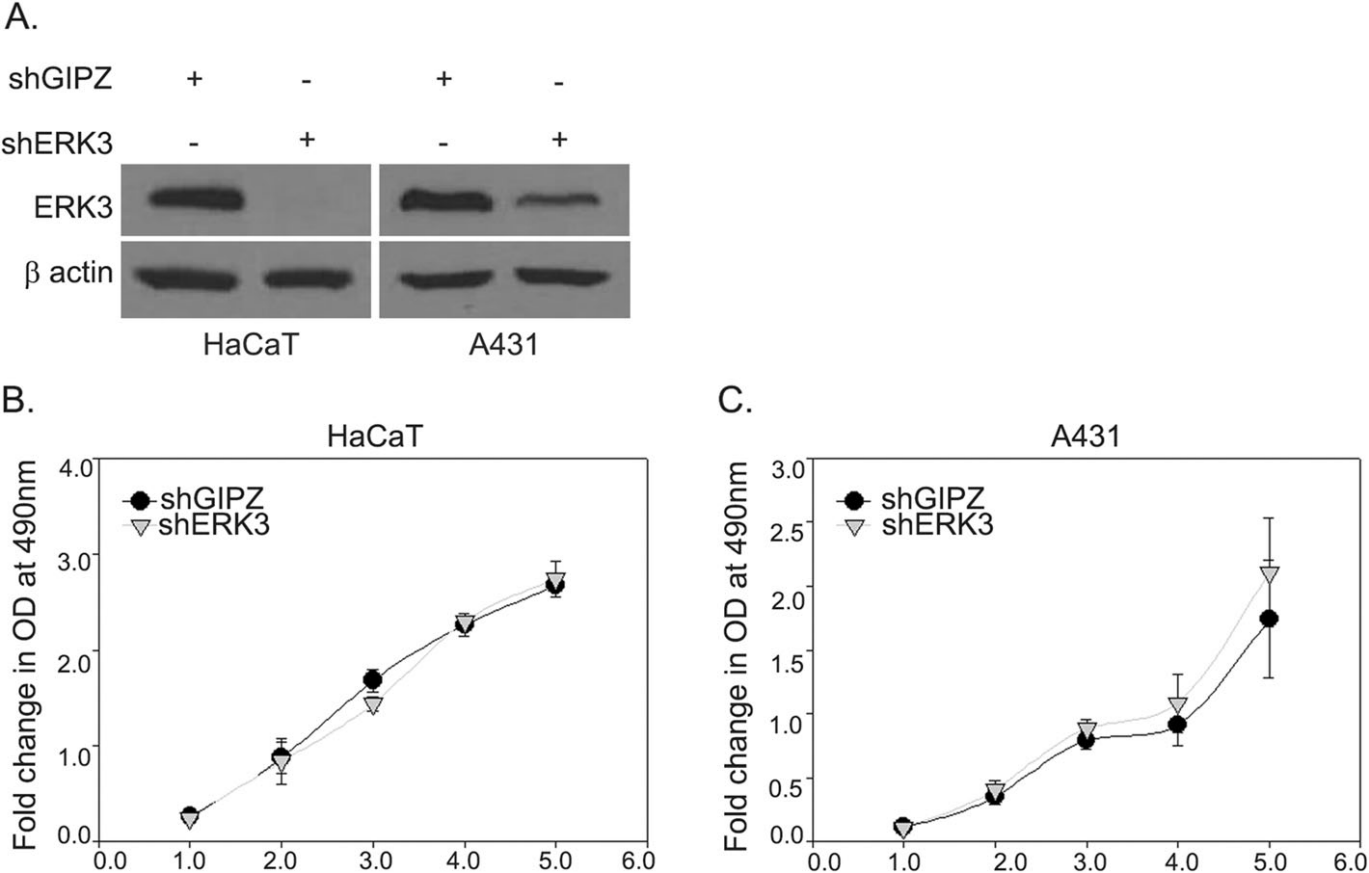
ERK3 knockdown does not affect cell proliferation. **a** HaCaT and A431 cells were stably transduced with lentiviruses expressing shRNA specifically targeting ERK3 (shERK3) or a non-targeting control shRNA (shGIPZ) as indicated. The change in protein levels of stable cells was analyzed by immunoblot analysis after 14 days of selection by puromycin. Representative western blots are shown as cropped gel images. Full length blots are presented in Additional File 5 (Figure S5). MTS cell proliferation assay was performed by measuring the number of viable cells daily for 5 days. The relative cell growth (indicated by optical density (OD) at 490 nm) of **b** HaCaT cells or **c** A431 cells expressing shERK3 or shGIPZ at different days are presented in the y-axis. Values represent mean ± S.D. The statistical analysis shows no significant difference between the shERK3 and the shGIPZ control in both cell lines as analyzed by Student’s *t*-test

ERK3 was shown to inhibit cell proliferation in multiple cancer cell lines, including a squamous cell carcinoma, hepatocarcinoma and melanoma cell lines [24, 36, 37]. In lung cancer cells, however, ERK3 does not impact cancer cell growth [18], indicating that the role of ERK3 role in cell growth is cancer-type dependent. Hence, we wanted to investigate whether ERK3 plays a role in cell growth in non-melanoma skin cancer cells. We generated HaCaT and A431 stable cell lines with stable knockdown of ERK3 by lentiviral transduction of shRNA against ERK3 (shERK3) or non-targeting lenti-GIPZ shRNA (shGIPZ) as a control. We confirmed the silencing of ERK3 in HaCaT-shERK3 or A431-shERK3 when compared with HaCaT-shGIPZ or A431-shGIPZ (Fig. 6a). As shown in Fig. 6b and c, ERK3 silencing did not cause a significant difference in proliferation between cells stably expressing shERK3 and cells expressing shGIPZ control in both HaCaT and A431 cell lines. This suggests that ERK3 does not affect cell proliferation in non-melanoma skin cancer cells.

### ERK3 regulates filopodia formation and decreases increased Rac1 phosphorylation upon ΔNp63α silencing

**Fig. 7:**
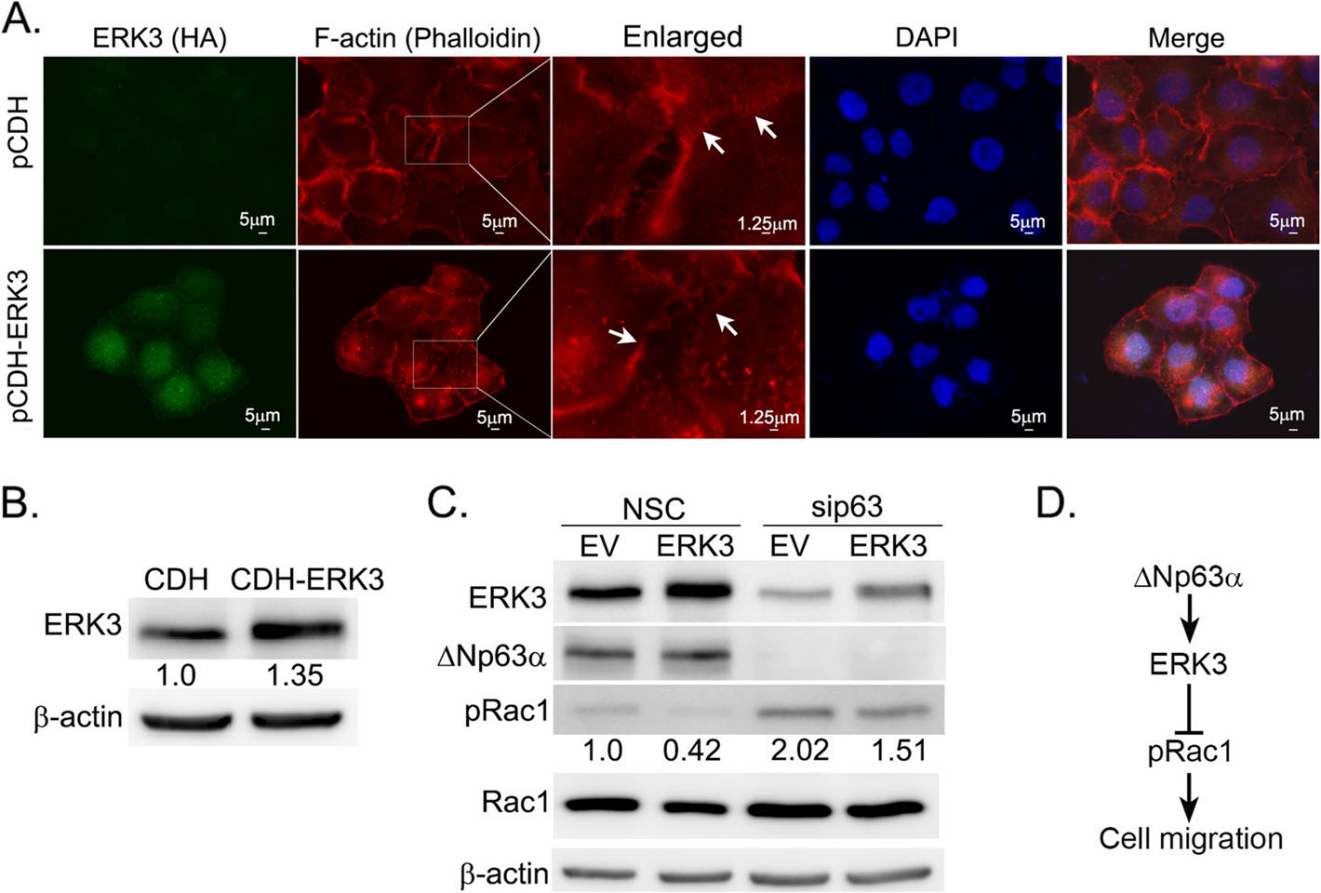
ERK3 overexpression inhibited filopodia formation and decreased Rac1 phosphorylation in A431 cells. A431 cells were transduced with lentiviral empty vector pCDH or pCDH-HA-ERK3 (pCDH-ERK3) for 48 h followed by **a** immunofluorescent detection of ERK3 using a HA antibody and F-actin (Phalloidin staining), or **b** immunoblot analysis to confirm the overexpression of ERK3. Arrows in **a** indicate the filopodia. Immunoblot with β-actin was performed to confirm equivalent protein loading. **c** A431 cells were transiently transfected for two rounds with non-silencing control siRNA (NSC) or ΔNp63α siRNA (sip63) as indicated. Along with the second siRNA transfection, cells were also transfected with empty vector control (EV) or HA-tagged ERK3 plasmid as indicated. 48 h post-transfection, cells were harvested, followed by Western blotting analysis of ERK3, ΔNp63α, pRac1 and total Rac1 protein. β-actin was immunoblotted as the loading control. Numbers below the pRac1 blot indicate the relative signal of pRac1 normalized by total Rac1 protein level and the β-actin level under each condition. The pRac1 signal in Lane 1 was arbitrarily set as 1. **d** A simple model of the ΔNp63α/ERK3 axis suppressing Rac1 phosphorylation and cell migration. Representative western blots are shown as cropped gel images. Full length blots are presented in Additional File 6 (Figure S6)

To investigate the mechanism by which ERK3 regulates cell migration in NMSC cells, we first examined whether ERK3 overexpression changes cell morphology. F-actin staining clearly showed that cells with ERK3 overexpression had a remarkable decrease in the formation of filopodia structures on cell surface and stress fibers assembly inside cells as compared to cells with expression of CDH empty vector (Fig. 7a and b), which is in line with its role of inhibiting cell migration in A431 cells. These results indicate that ERK3 inhibits filopodia formation during cell migration.

We have previously reported that ΔNp63α inhibits cell migration and invasion via downregulating Rac1 phosphorylation [29]. These findings prompted us to examine whether ΔNp63α-ERK3 signaling downregulates cell migration through inhibiting Rac1 phosphorylation. We examined the change in Rac1 phosphorylation upon transfecting A431 cells with non-targeting siControl or sip63 along with the overexpression of the empty vector or ERK3 plasmid. Indeed, overexpression of ERK3 reduced Rac1 phosphorylation (pRac1 blot, Lane 2 versus Lane 1, Fig. 7c). As expected, silencing ΔNp63α increased pRac1 (Lane 3 versus Lane 1, Fig. 7c). Importantly, ERK3 overexpression counteracted the increase of pRac1 induced by silencing ΔNp63α (Lane 4 versus Lane 3, Fig. 7c). These results suggest that ΔNp63α -ERK3 axis inhibits Rac1 phosphorylation, thereby suppressing NMSC cell migration.

## Discussion

ΔNp63α plays an important role in the development of skin, lung and mammary gland [3, 38, 39]. ΔNp63α is overexpressed in many human cancers, such as skin cancer, head and neck cancers, lung cancers, and esophageal squamous cell carcinomas [6, 40–42]. ΔNp63α exhibits an inhibitory role on cell migration and invasion, in part by downregulating EMT and Akt pathway [10, 11, 29, 34, 35, 43, 44]. Hence, the role of ΔNp63α in cancer development and progression is complex and seems to be tissue-type dependent. The underlying molecular mechanisms by which ΔNp63α plays different roles in different cancers are still largely unknown and need to be further explored.

ERK3 has been shown to be an essential player in organogenesis and cancer cell growth and invasiveness [17, 45]. ERK3 overexpression has been found in several human cancers, including squamous cell lung carcinoma, oral squamous cell carcinoma, gastric cancer, breast cancer and melanoma [18–21, 23]. ERK3 promotes cancer cell migration and invasion but has little effect on the proliferation of several types of human cancer cells, including those of lung, breast, and head and neck cancers. A recent study showed that ERK3 was reported to inhibit melanoma cell migration and proliferation [24]. Hence, like ΔNp63α, ERK3 is upregulated in lung SCC and head and neck SCC and plays differential roles in different cancers. However, there is no report about the involvement of ERK3 in cutaneous SCC.

In this study, we showed that ERK3 protein is expressed in normal skin and non- melanoma skin tissues, including AK, SCC and BCC. Consistent with the findings of previous studies including those from our own laboratory [6, 10], ΔNp63α expression level is elevated in AK, SCC, and BCC. This is the first report that ERK3 protein is upregulated in cutaneous SCCs. Interestingly, unlike ΔNp63α, ERK3 expression in cutaneous BCC did not show a significant difference from that of the normal skin tissue. More importantly, our results demonstrate a highly significant positive correlation between ΔNp63α expression and ERK3 expression in normal skin and NMSC specimens. These findings raise an intriguing possibility that ΔNp63α, as a transcriptional factor, may regulate the expression of ERK3 in skin and indicates a cooperative role between ΔNp63α and ERK3 in promoting the initiation and development of cutaneous SCC. Indeed, we have found that ΔNp63α positively regulates ERK3 gene expression in both HaCaT keratinocyte and A431 skin SCC cells.

The inhibitory role of ΔNp63α on cell migration is attributed to its function in sustaining the epithelial integrity by blocking pathways that promote EMT. Interestingly, similar to ΔNp63α, ERK3 is also involved in cell migration and invasion and exhibits different roles in different types of cancer cells. While it promotes migration of lung cancer cells and breast cancer cells [18, 21], ERK3 inhibits migration of melanoma cell lines [24]. In this study, we found that knockdown of ERK3 significantly increased the migration of A431 cutaneous SCC cells, suggesting that both ΔNp63α and ERK3 play inhibitory roles in skin cell migration. Importantly, restoring ERK3 expression in A431 cells counteracted the increase in cell migration due to the depletion of ΔNp63α. Mechanistically, ERK3 overexpression inhibits Rac1 phosphorylation and the formation of filopodia in A431 cells. This indicates that the inhibitory role of ΔNp63a-ERK3 axis on cell migration is at least partly via downregulating Rac1 phosphorylation (Fig. 7d). However, we cannot rule out the possibility of the involvement of other downstream targets in this process. Taken together, these findings demonstrate that ERK3 is a mediator for ΔNp63α-mediated inhibition of NMSC cell migration.

## Conclusion

In summary, we have found that ERK3 is upregulated in cutaneous SCC and its expression level is positively correlated with ΔNp63α. In line with these clinical findings, ERK3 transcript levels are positively regulated by ΔNp63α, and ERK3 acts as an important downstream mediator of ΔNp63α in regulating cell migration. To our knowledge, our study is the first to reveal the molecular regulation of ERK3 by ΔNp63α in cutaneous SCC and provides an additional mechanism by which ΔNp63α regulates cancer cell migration. As both ΔNp63α and ERK3 are upregulated in lung squamous cell carcinomas, future investigations are required to determine the interplay between ΔNp63α and ERK3 in this type of cancer.

## Supplementary Information

**Additional file 1: Figure S1.** Representative images of Normal, AK, SCC and BCC skin tissues after Haemotoxylin and Eosin (H&E) staining.

**Additional file 2: Figure S2.** Full-length Western blots for Fig. 3c.

**Additional file 3: Figure S3.** Full-length Western blots for Fig. 4a.

**Additional file 4: Figure S4.** Full-length Western blots for Fig. 5a. In each image, lane 1 corresponds to the lysate of the NSC and CDH expression, lane 2 to sip63 and CDH, and lane 3 to sip63 and CDH-ERK3.

**Additional file 5: Figure S5.** Full-length Western blots for Fig. 6a.

**Additional file 6: Figure S6.** Full-length Western blots for Fig. 7b and c.

**Additional file 7: Table S1.** Descriptive statistics for ΔNp63α and ERK3 co-immunofluorescence staining in normal skin and non-melanoma cancer tissue microarrays. Normal skin (*N* = 53), cutaneous squamous cell carcinoma (SCC) (*N* = 59), basal cell carcinoma of the skin (BCC) (*N* = 57), and actinic keratosis (*N* = 66) tissue microarrays sections were immunostained for ΔNp63α and ERK3. “*n Samples*” refers to the number of tissue samples whereas “*n Obs*” refers to the total number of observations. The means are given as least squares means that control for an imbalanced sample size (not all samples have nine observations).

**Additional file 8: Table S2.** Dunnett’s test for ΔNp63α and ERK3 co-immunofluorescence staining in normal skin and non-melanoma cancer tissue microarrays. Based on *P*-values (are less than alpha = 0.05), there is strong evidence to suggest that the mean MFI for ΔNp63α is significantly different between normal skin tissue and BCC of the skin tissue, normal skin tissue and cutaneous SCC tissue, and normal skin tissue and AK of the skin tissue (*P*-values of < 0.0001, 0.0015, and < 0.0001, respectively). Since all the estimated differences are positive, we can infer that ΔNp63α is upregulated in BCC, SCC, and AK of the skin tissue relative to normal skin tissue. The estimated mean differences were 21.09 MFI higher for BCC [95% confidence interval of (14.54, 27.64)], 9.70 MFI higher for SCC [95% confidence interval of (3.21, 16.19)] and 27.19 MFI higher for AK [95% confidence interval of (20.86, 33.52)].

**Additional file 9: Table S3.** Correlation for the mean fluorescence intensity of ΔNp63α and ERK3 in each skin tissue type.

## Data Availability

Original data can be available from the corresponding author upon reasonable request.

## Abbreviations

NMSC: Non-melanoma skin cancer
ERK3: Extracellular signal-regulated kinase 3
SCC: Squamous cell carcinoma
siRNA: Small interfering RNA
shRNA: Short hairpin RNA
NSC: Non-silencing control
