# Supplementary material for: ERK3 is transcriptionally upregulated by ∆Np63α and mediates the role of ∆Np63α in suppressing cell migration in non-melanoma skin cancers
Source: BMC Cancer. 2021 Feb 12;21:155. doi: 10.1186/s12885-021-07866-w (PMC7881562; doi:10.1186/s12885-021-07866-w)

Additional file 1: Fig. S1 Representative images of Normal, AK, SCC and BCC skin after Haemotoxylin and Eosin (H&E) staining.

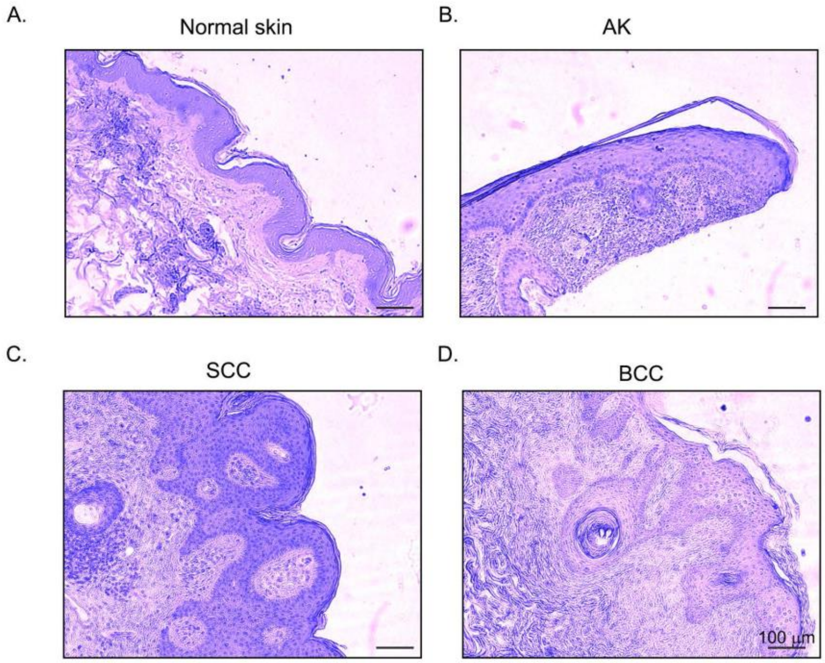

Supplement: Supplementary file 1 — Additional file 1: Figure S1. Representative images of Normal, AK, SCC and BCC skin tissues after Haemotoxylin and Eosin (H&E) staining. [file 12885_2021_7866_MOESM1_ESM.pdf]
