# Supplementary material for: ERK3 is transcriptionally upregulated by ∆Np63α and mediates the role of ∆Np63α in suppressing cell migration in non-melanoma skin cancers
Source: BMC Cancer. 2021 Feb 12;21:155. doi: 10.1186/s12885-021-07866-w (PMC7881562; doi:10.1186/s12885-021-07866-w)

Additional file 2: Figure S2. Full-length Western blots for Figure 3C

Full length blots for figure 3C A431 cells:

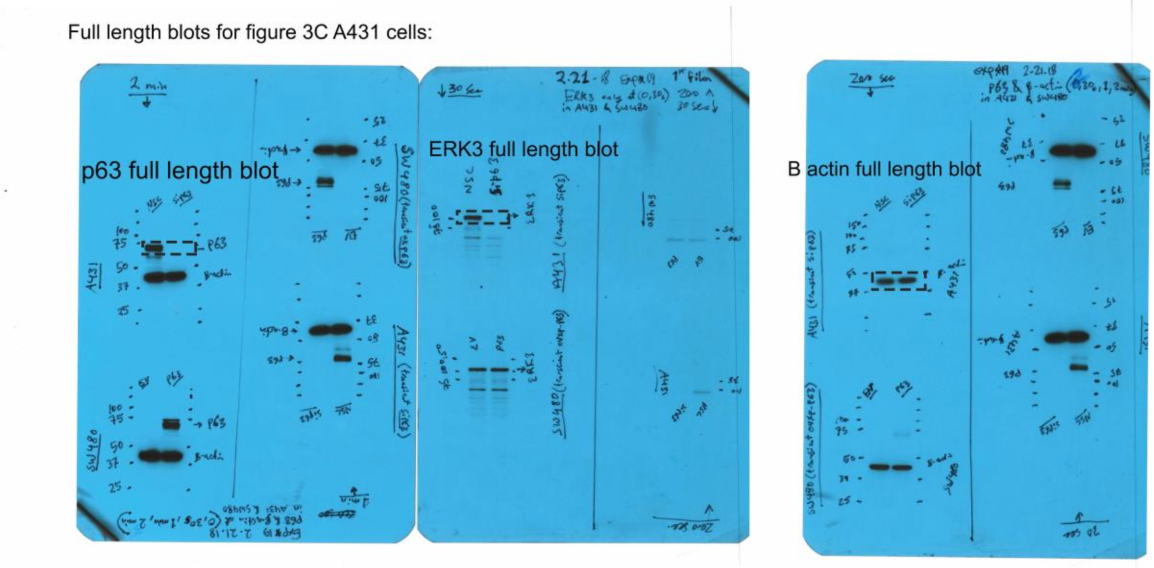

Full length blots for figure 3C HaCaT cells:

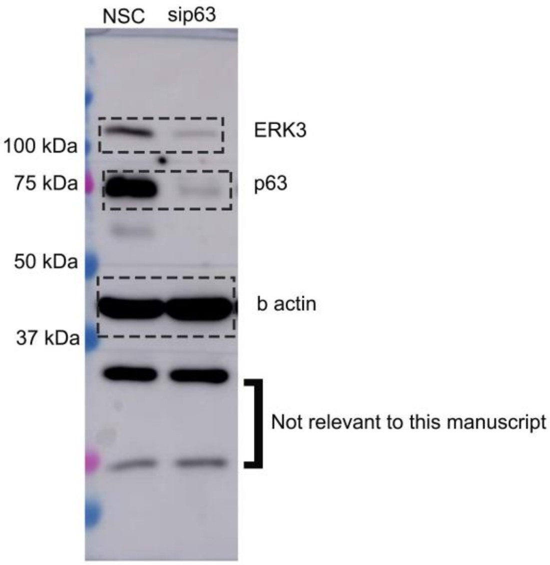

Supplement: Supplementary file 2 — Additional file 2: Figure S2. Full-length Western blots for Fig. 3c. [file 12885_2021_7866_MOESM2_ESM.pdf]
