# Supplementary figures and images for: ERK3 is transcriptionally upregulated by ∆Np63α and mediates the role of ∆Np63α in suppressing cell migration in non-melanoma skin cancers
Source: BMC Cancer. 2021 Feb 12;21:155. doi: 10.1186/s12885-021-07866-w (PMC7881562; doi:10.1186/s12885-021-07866-w)

Additional file 3: Figure S3. Full-length Western blots for Figure 4A

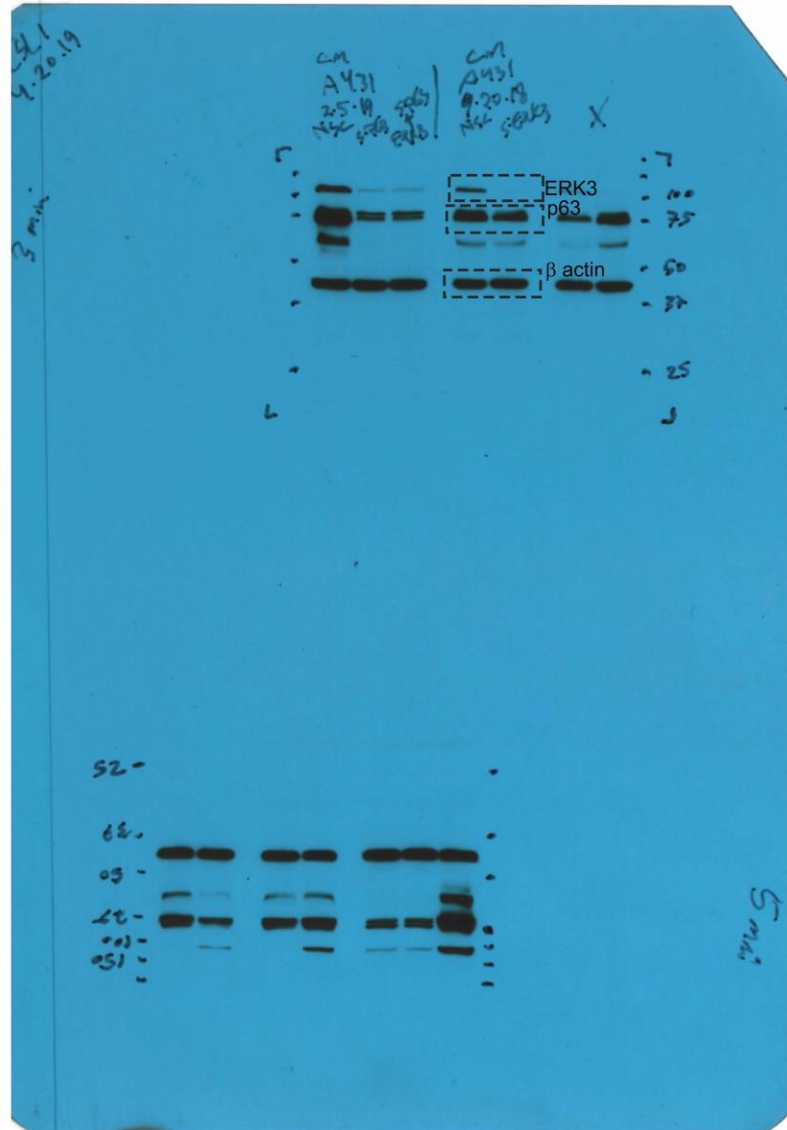

Supplement: Supplementary file 3 — Additional file 3: Figure S3. Full-length Western blots for Fig. 4a. [file 12885_2021_7866_MOESM3_ESM.pdf]

A.

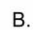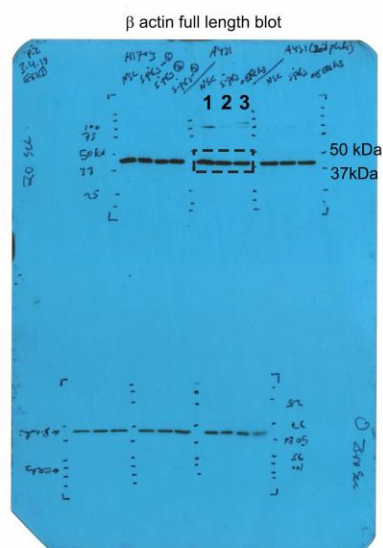

Supplement: Supplementary file 4 — Additional file 4: Figure S4. Full-length Western blots for Fig. 5a. In each image, lane 1 corresponds to the lysate of the NSC and CDH expression, lane 2 to sip63 and CDH, and lane 3 to sip63 and CDH-ERK3. [file 12885_2021_7866_MOESM4_ESM.pdf]

Additional file 5: Figure S5. Full-length Western blots for Figure 6A

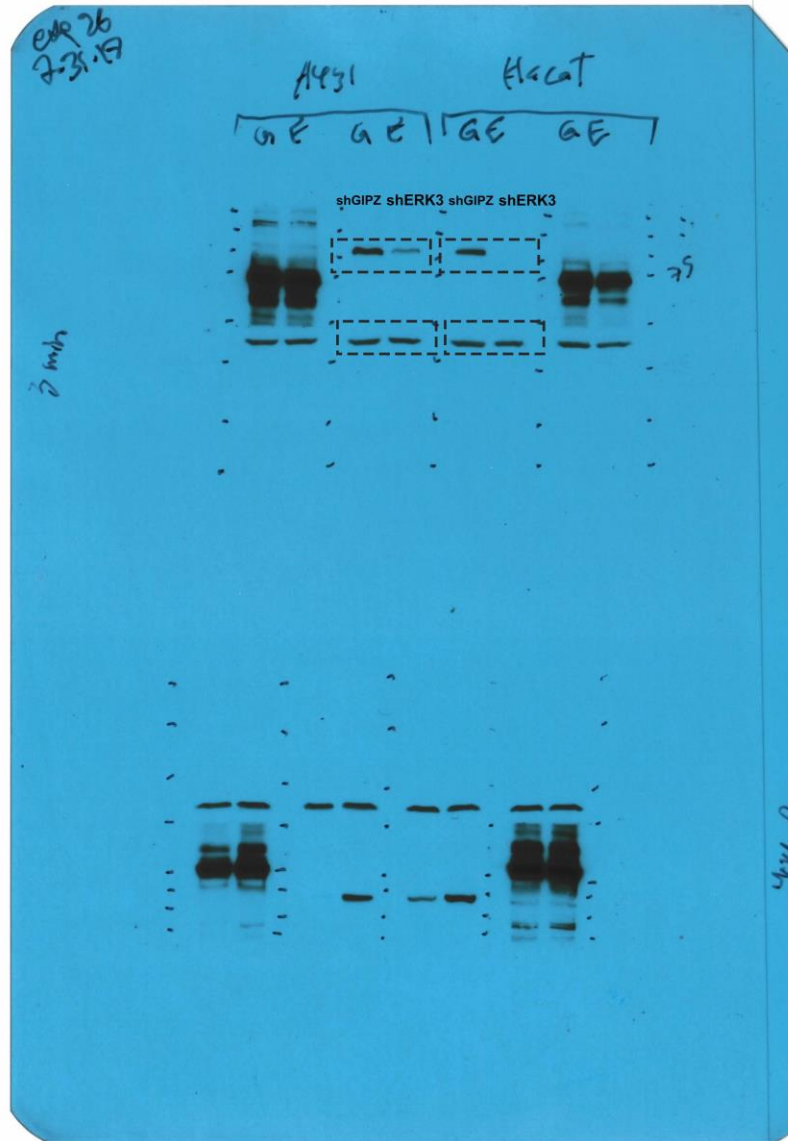

Supplement: Supplementary file 5 — Additional file 5: Figure S5. Full-length Western blots for Fig. 6a. [file 12885_2021_7866_MOESM5_ESM.pdf]

Additional file 6: Figure S6. Full-length Western blots for Figure 7B and 7C

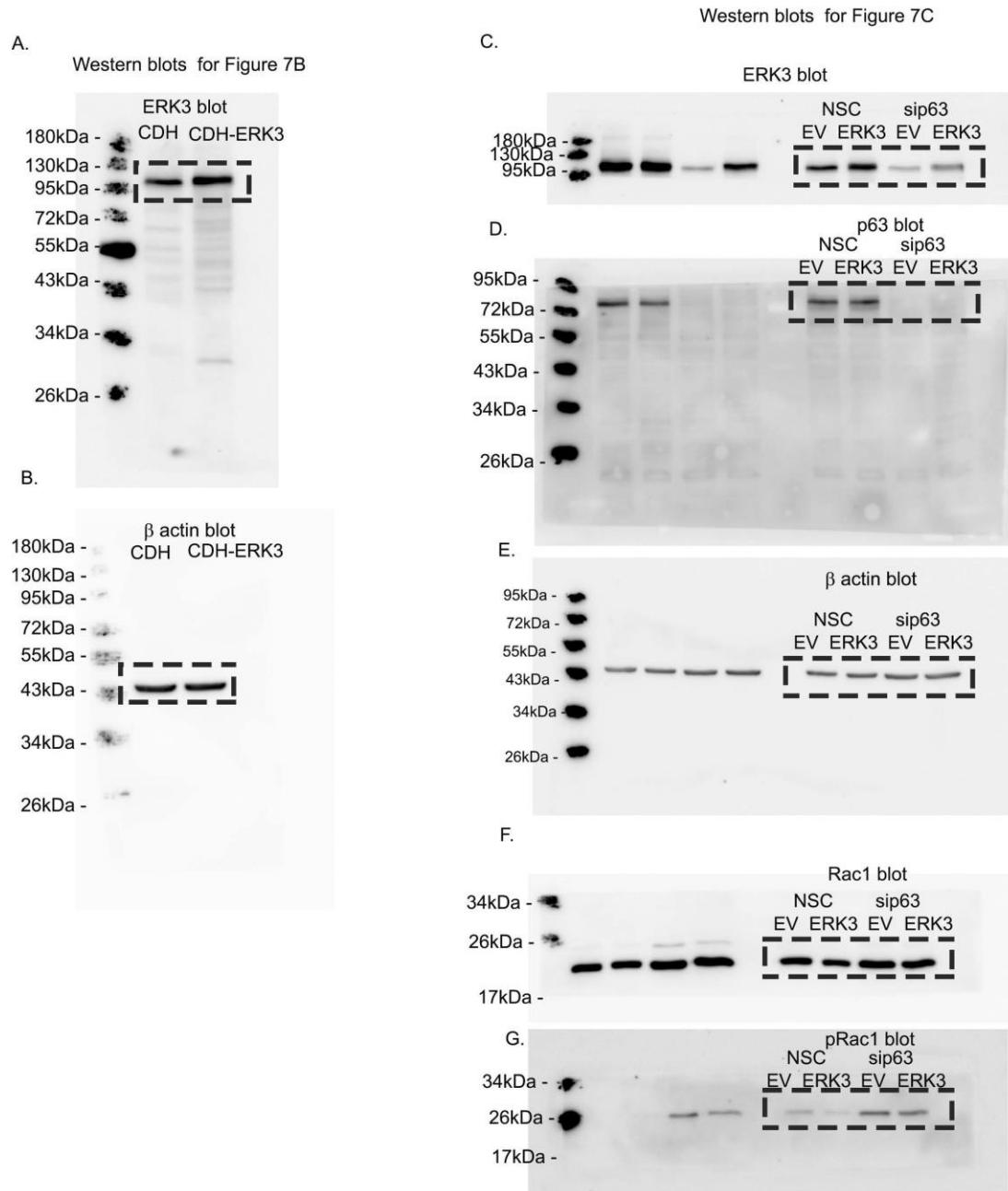

Supplement: Supplementary file 6 — Additional file 6: Figure S6. Full-length Western blots for Fig. 7b and c. [file 12885_2021_7866_MOESM6_ESM.pdf]
