# Supplementary material for: ERK3 is transcriptionally upregulated by ∆Np63α and mediates the role of ∆Np63α in suppressing cell migration in non-melanoma skin cancers
Source: BMC Cancer. 2021 Feb 12;21:155. doi: 10.1186/s12885-021-07866-w (PMC7881562; doi:10.1186/s12885-021-07866-w)
Supplement: Supplementary file 7 — Additional file 7: Table S1. Descriptive statistics for ΔNp63α and ERK3 co-immunofluorescence staining in normal skin and non-melanoma cancer tissue microarrays. Normal skin (N = 53), cutaneous squamous cell carcinoma (SCC) (N = 59), basal cell carcinoma of the skin (BCC) (N = 57), and actinic keratosis (N = 66) tissue microarrays sections were immunostained for ΔNp63α and ERK3. “n Samples” refers to the number of tissue samples whereas “n Obs” refers to the total number of observations. The means are given as least squares means that control for an imbalanced sample size (not all samples have nine observations). [file 12885_2021_7866_MOESM7_ESM.pdf]

Additional file 7: Table S1.

Descriptive Statistics for p63 MFI

Type	*n Samples*	*n Obs*	*Least Squares Mean*	*Standard Error*
Normal	53	474	31.49	2.00
BCC	57	507	52.58	1.93
SCC	59	531	41.19	1.89
AK	66	576	58.68	1.79

Descriptive Statistics for ERK3 MFI

Type	*n Samples*	*n Obs*	*Least Squares Mean*	*Standard Error*
Normal	53	474	23.34	1.62
BCC	57	507	26.95	1.56
SCC	59	531	31.21	1.54
AK	66	576	39.81	1.45
