# Supplementary material for: ERK3 is transcriptionally upregulated by ∆Np63α and mediates the role of ∆Np63α in suppressing cell migration in non-melanoma skin cancers
Source: BMC Cancer. 2021 Feb 12;21:155. doi: 10.1186/s12885-021-07866-w (PMC7881562; doi:10.1186/s12885-021-07866-w)
Supplement: Supplementary file 8 — Additional file 8: Table S2. Dunnett’s test for ΔNp63α and ERK3 co-immunofluorescence staining in normal skin and non-melanoma cancer tissue microarrays. Based on P-values (are less than alpha = 0.05), there is strong evidence to suggest that the mean MFI for ΔNp63α is significantly different between normal skin tissue and BCC of the skin tissue, normal skin tissue and cutaneous SCC tissue, and normal skin tissue and AK of the skin tissue (P-values of < 0.0001, 0.0015, and < 0.0001, respectively). Since all the estimated differences are positive, we can infer that ΔNp63α is upregulated in BCC, SCC, and AK of the skin tissue relative to normal skin tissue. The estimated mean differences were 21.09 MFI higher for BCC [95% confidence interval of (14.54, 27.64)], 9.70 MFI higher for SCC [95% confidence interval of (3.21, 16.19)] and 27.19 MFI higher for AK [95% confidence interval of (20.86, 33.52)]. [file 12885_2021_7866_MOESM8_ESM.pdf]

Additional file 8: Table S2.

Results of Dunnett's Test for p63 MFI

Comparison	*Difference*	*Adj. P-value*	*Adj. 95% Confidence Interval*	*Fold Change*
BCC – Normal	21.09	< 0.0001	(14.54, 27.64)	1.67
SCC – Normal	9.70	0.0015	(3.21, 16.19)	1.31
AK – Normal	27.19	< 0.0001	(20.86, 33.52)	1.86

Results of Dunnett's Test for ERK3 MFI

Comparison	*Difference*	*Adj. P-value*	*Adj. 95% Confidence Interval*	*Fold Change*
BCC – Normal	3.61	0.25	(-1.70, 8.92)	1.15
SCC – Normal	7.87	0.0015	(2.60, 13.14)	1.34
AK – Normal	16.47	< 0.0001	(11.33, 21.61)	1.71
