# Supplementary material for: ERK3 is transcriptionally upregulated by ∆Np63α and mediates the role of ∆Np63α in suppressing cell migration in non-melanoma skin cancers
Source: BMC Cancer. 2021 Feb 12;21:155. doi: 10.1186/s12885-021-07866-w (PMC7881562; doi:10.1186/s12885-021-07866-w)
Supplement: Supplementary file 9 — Additional file 9: Table S3. Correlation for the mean fluorescence intensity of ΔNp63α and ERK3 in each skin tissue type. [file 12885_2021_7866_MOESM9_ESM.pdf]

Additional file 9: Table S3.
Correlations for p63 and ERK3 MFI within each Skin Tissue Type

Type	*Estimate*	*Standard Error*	*95% Confidence Interval*	*P-value*
Normal	0.79	0.04	(0.72, 0.86)	< 0.0001
BCC	0.63	0.05	(0.52. 0.73)	< 0.0001
SCC	0.78	0.04	(0.71, 0.85)	< 0.0001
AK	0.74	0.03	(0.67, 0.81)	< 0.0001
